# Comment on “Effects of *in Utero* Exposure to Arsenic during the Second Half of Gestation on Reproductive End Points and Metabolic Parameters in Female CD-1 Mice”

**DOI:** 10.1289/ehp.1511031

**Published:** 2016-03-01

**Authors:** Amy L. Williams, John M. DeSesso

**Affiliations:** Exponent, Inc., Alexandria, Virginia, USA

Rodriguez et al. recently reported that the adult female offspring of pregnant CD-1 mice exposed to 10 ppb or 42.5 ppm arsenic (As) exhibited reproductive and metabolic effects. These findings are not consistent with those of others working in this field and did not show a dose response. As such, we urge caution against drawing conclusions based on this single study.

Rodriguez et al. reported that on postnatal day 21, female offspring in the 10-ppb and 42.5-ppm dose groups were approximately 11% and 7% heavier, respectively, than controls. At 6 months of age, the treated female offspring were ≥ 22% (the exact percentages are unclear from the study report) heavier than controls. The study authors mention data from other investigators using C57BL6/J mice showing no effects of gestational As exposure on offspring body weight (Ramsey et al. 2013; Kozul-Horvath et al. 2012). Other studies using a similar exposure paradigm also reported no effects on body weight of the female offspring of C3H mice (Waalkes et al. 2003, 2004), C57BL6/J mice (Markowski et al. 2011, 2012), or Tg.AC mice (Tokar et al. 2010).

The authors suggest that discrepancies between their results and those of others may be due to differences in the genetic background of the mice in the various studies. However, Waalkes et al. (2006) conducted a study in pregnant CD-1 mice exposed to 0 or 85 ppm sodium arsenite from gestational day (GD) 8 to GD18 with no effects of treatment on female offspring body weight. In another study (Tokar et al. 2011) involving whole-life exposure (from preconception into adulthood) of CD-1 mice to 6, 12, or 24 ppm sodium arsenite, body weights of treated mice were similar to those of controls at all time points assessed. It is also interesting to note that controls in the study by Tokar et al. (2011) weighed considerably more (42.4 g at 25 weeks of age) than controls in the Rodriguez et al. study (approximately 34.4 g at 26 weeks of age).

The reason for this discrepancy between the findings of Rodriguez et al. and those of other investigators is not known but may relate to differences in diet or husbandry. Alternatively, it is possible that the controls in the study by Rodriguez et al. are unusually small for their age, such that the observed effect of treatment may be a statistical anomaly; this could explain why a dose-related difference was not observed.

Rodriguez et al. also reported that both doses of As were associated with early vaginal opening; again, no dose response was evident. The authors mention the results of two other studies in their discussion—both of which showed delays in puberty rather than early onset (Reilly et al. 2014; Davila-Esqueda et al. 2012). We identified two other studies that examined vaginal opening with gestational-only As exposure. Markowski et al. (2012) exposed pregnant C57BL6/J mice to 0, 8, 25, or 80 ppm sodium arsenite from GD4 until birth with no effects on the onset of puberty. Gandhi et al. (2012) exposed pregnant albino rats to 0, 1.5, 3, or 4.5 mg As/kg/day from GD8 until birth; again, no effects on vaginal opening were observed.

The onset of puberty is positively correlated with body weight (Carney et al. 2004). Unfortunately, Rodriguez et al. did not report the mean weights at vaginal opening; therefore, we do not know if the early vaginal opening in the As-treated groups may have been a function of the increased body weights. However, animals in the 10-ppb and 42.5-ppm groups weighed more than controls and thus were likely to reach puberty earlier than controls.

In closing, the findings of Rodriguez et al. with regard to body weight and pubertal effects conflict with those of other investigators. Until other investigators can replicate the results reported by Rodriguez et al., we believe these findings should be viewed with extreme caution.
